# Age-related disturbances in rest-activity rhythms and integrity of the hippocampal network: An exploratory study

**DOI:** 10.1016/j.nbscr.2024.100111

**Published:** 2024-12-26

**Authors:** Aurore Jouvencel, Bixente Dilharreguy, Marion Baillet, Karine Pérès, Jean-François Dartigues, Hélène Amieva, Willy Mayo, Gwenaëlle Catheline

**Affiliations:** a INCIA, EPHE, Université PSL, Univ Bordeaux, CNRS, 146, Rue Léo Saignat, 33076, Bordeaux, France; b Athinoula A. Martinos Center for Biomedical Imaging, Department of Radiology, Massachusetts General Hospital and Harvard Medical School, Boston, MA, USA; c INSERM, Bordeaux Population Health Research Center, University of Bordeaux, UMR U1219, 146, Rue Léo Saignat, 33076, Bordeaux, France

**Keywords:** Rest-activity rhythms, Hippocampal atrophy, Longitudinal, MRI, Aging

## Abstract

To better understand the relationship between the rest-activity rhythms and cognitive impairments during aging, we assessed the longitudinal changes in the rest-activity rhythms in an elderly population and their possible detrimental effect on the hippocampal network.

This was done longitudinally in a rural cohort with two actigraphic assessments and brain imaging examinations, seven years apart. A segmentation of the hippocampus and its related structures was used to assess volumes and functional connectivity in this network based on anatomical and resting state functional data. Regression models were carried out to investigate the potential association of the evolution of sleep and rest-activity rhythms parameters with the structural and functional integrity of the hippocampal network.

Our sample was composed of 33 subjects aged 75.2 ± 2.4 years old at the first time point with 40% of women. After seven years, the sleep of our participants did not change but their rest-activity rhythms did (p < 0.05), with a decrease in relative amplitude (∂RA = −0.021) and stability (∂IS = −0.044) as well as an increase in fragmentation (∂IV = +0.072). The deterioration of rest-activity rhythms was correlated with a lower anterior hippocampal volume (p corrected <0.05) while no correlation with functional connectivity was observed.

These findings suggest that a degradation of rest-activity rhythms in people over 70 years old could constitute a factor of hippocampal vulnerability. Preventive interventions should consider rest-activity rhythms in the oldest-old population.

## Highlights

• After 75 years of age, sleep does not change while the sleep-wake cycle degrades.
• Sleep-wake cycle degradation is linked to smaller anterior hippocampus volume.
• Sleep-wake cycle degradation could lead to loss of homeostasis preservation.

## Graphical abstract

**Figure:**
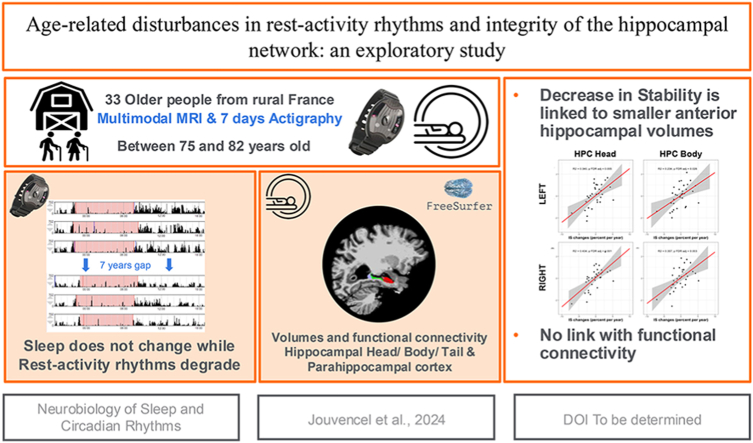

## 1 Introduction

Sleep has been widely studied in the aging population, in particular regarding its association with cognitive abilities (Dzierzewski et al., 2018; Leong and Chee, 2023; Mander et al., 2017; Rasch and Born, 2013; Van Someren et al., 2015). But over the 24-h period, sleep is not the only state to be expressed and alternates with wakefulness to form the rest-activity rhythms measured through the rest-activity rhythm with actigraphy. In addition to sleep, which have received most attention so far, the rest-activity rhythms is also affected during aging (Luik et al., 2013). In 144 home-dwelling older participants (aged 69.5 ± 8.5), the rest-activity rhythms fragmentation was negatively associated with mental speed, executive function and memory (Oosterman et al., 2009). In a recent review, Carlson et al., bring together a number of animal and human studies suggesting that hippocampal-dependent memory processes can be adversely affected by a disruption in circadian processes independently of sleep (Carlson et al., 2023). And to date, there is only one study that has measured the evolution of the rest-activity rhythms in a longitudinal setting (Li et al., 2020). In this study, they followed a large sample of the American population over 59 years old (1401 participants) for 15 years with regular actigraphic measurements. They found a degradation of the rest-activity rhythms through a decrease in amplitude, acrophase and inter-daily stability as well as an increase in intra-daily variability (i.e. fragmentation). Those degradations were linked to a decrease in global cognition as well as a higher risk of developing Alzheimer's disease (AD). In turn, AD progression accelerated the degradation of the rest-activity rhythms in their population (Li et al., 2020). The link between the evolution of the rest-activity rhythms and AD seems to be bi-directional. But the question remains, what are the physiological mechanisms underlying the link between the rest-activity rhythms and dementia?

One possibility is that those rest-activity rhythms impairments have adverse effect on hippocampal memory network, in the form of structural and/or functional changes. Hippocampal atrophy is present in normal aging (Nadal et al., 2020) and in AD (Jack et al., 2013). Multiple task-based functional imaging studies showed a link between sleep and global hippocampal activity (Jonelis et al., 2012; Van Der Werf et al., 2009; Yoo et al., 2007). But the hippocampus is not a homogenous structure, it is made of different subregions (Genon et al., 2021) and is part of a wider temporal network, including the Entorhinal cortex, Medial and Lateral Para-Hippocampal cortex (Huijgen and Samson, 2015). Functional imaging studies reported a long-axis functional specialization of the hippocampus, which arises from anatomical and electrophysiological differences such as large-scale network connectivity (Genon et al., 2021). Considering functional connectivity at rest, in a data-driven analysis, Chang et al. found that the hippocampus shows a longitudinal axis organization that is not comparable to the lamellar structure (Chang et al., 2021). In order to consider not only hippocampal volumes but also hippocampal resting-state functional connectivity (Zajac et al., 2020), a head-body-tail segmentation of the hippocampus seems more suitable to jointly investigate the structural and functional state of this structure.

In this exploratory study, we hypothesize that the degradation of sleep and rest-activity rhythms in “typical” aging could predict the structural and functional state of the hippocampal network. This detrimental effect of the rest-activity rhythms degradation on hippocampal network could partly explain the high prevalence of AD in individuals presenting rest-activity rhythms disturbances. We tested this hypothesis in a cohort of cognitively healthy French older participants (>70 years old) that have been included in a longitudinal study with two actigraphic measures and Magnetic Resonance Imaging (MRI) scans, seven years apart on average. We examined hippocampal network state through volumes and functional connectivity at rest within the different regions of interest (hippocampus, entorhinal cortex and para-hippocampal cortex).

## 2 Methods

### 2.1 Population

This study is part of AMImage, an ancillary research of the Aging Multidisciplinary Investigation (AMI) cohort (Pérès et al., 2012), which aims at studying cerebral and functional aging in rural area. AMI is an epidemiological prospective study started in 2007, which includes individuals older than 65 years old living in the Gironde region (France) and who have been working for more than 20 years in the agricultural field. At each follow-up (every 2–3 years), participants were visited at home by a neuropsychologist to administer a complete battery of neuropsychological tests and collect a large panel of data (sociodemographic, lifestyle, health …). The following factors have been analyzed in the present study. Body mass index (BMI) was assessed, and educational level was categorized in three levels (primary school or less; high school; and university). The presence of at least one ε4 allele of the apolipoprotein E (APOE ε4) gene was derived from blood samples. The Mini Mental State Examination (MMSE) was used to evaluate global cognitive status (Folstein et al., 1975). Sleep apnea diagnosis was self-reported. Consumption of psycholeptic drugs was collected, such as anxiolytics, hypnotics and/or sedatives, and antipsychotics for anxiety as well as hypertension medications.

This study procedure was approved by a regional human research review board (Code AMI2: 2011-A01393-38; AMI3: 2019-A00253-54) and all participants provided written informed consent.

In the AMImage study, an MRI scan was proposed to a sub-sample of AMI participants on three occasions (2009–2011; 2012–2014; 2019–2022). All AMImage participants were right-handed and had no neurological or psychiatric disorders or any MRI contraindications. During the second and third scans (AMI2 and AMI3), participants wore an actigraphy device for a week. The present study focused on the 40 participants having done AMI2 and AMI3 with the actigraphy assessments. Among them, five were diagnosed with AD after AMI3; one had a stroke, and one had generalized brain atrophy with cognitive impairment. The study sample is composed of 33 healthy persons.

### 2.2 Actigraphy assessment

Sleep and rest-activity rhythms were measured with wrist-worn actigraphs, the ActiWatch 7 and the MotionWatch 8 (Cambridge Neurotechnology, Cambridge, UK), validated against polysomnography (Kushida et al., 2001; Sadeh, 2011). The MotionWatch8 were used with the MotionWatch Mode 1 that replicate the set-up of the ActiWatch7. The devices were placed on the nondominant wrist and were kept continuously for a week in the home environment. MotionWare, v1.2.26 (Cambridge Neurotechnology, Cambridge, UK) with a sensitivity threshold of 20 counts was used to process the actigraphy data. A sleep diary informing about bedtime and rise time was completed by each participant during the protocol and was used to improve data scoring.

Sleep and rest-activity rhythms parameters studied in the analyses are presented in Table 1, regarding rest-activity rhythms, a Non-Parametric Circadian Rhythm Analysis (NPCRA) was used.

**Table 1:** Actigraphic parameters.

Definitions	Abbreviations
*Sleep parameters* (Fekedulegn et al., 2020)	

Time In Bed	TIB
Total Sleep Time	TST
Wake After Sleep Onset	WASO
Sleep Efficiency	SE
Sleep Fragmentation	SF

*Rest-activity cycle parameters* (Van Someren et al., 1999)	

Relative Amplitude	RA
Intra-daily Variability: the degree of fragmentation of activity-rest periods	IV
Inter-daily Stability: the degree of regularity in the activity-rest pattern.	IS

### 2.3 MRI acquisition and analysis

AMI2 and AMI3 morphologic MRI acquisition.

AMI2 MRI data were acquired using an Achieva 3T scanner (Philips Medical System, The Netherlands) with a SENSE 8-channel head coil. A high-resolution T1-weighted (T1w) structural scan was acquired with a three-dimensional (3D) magnetization-prepared rapid acquisition gradient echo (MPRAGE) sequence with the following parameters: repetition time (TR) = 8.2 ms, echo time (TE) = 3.5 ms, flip angle (FA) = 7°, field of view (FOV) of 256 × 256 × 180 mm^3^ with a voxel size of 1 mm^3^. Additional 3D T2-weighted (T2w) images were acquired with the following parameters: TR = 2500 ms, TE = 363 ms, FA = 90°, FOV of 256 × 256 × 180 mm^3^ with a voxel size of 1 mm^3^.

AMI3 MRI data were acquired using a Siemens Prisma 3T scanner (Siemens Healthcare, Germany) equipped with a 64-channel head/neck coil. A high-resolution T1w structural scan was acquired with a 3D MPRAGE sequence (TR = 2120 ms; TE = 2.35 ms; FA = 9°; Inversion Time = 1070 ms; FOV of 256x256 × 192 mm^3^ with a voxel size of 0.8 mm^3^). Additional 3D T2w images were acquired with the following parameters: TR = 2600 ms, TE = 161 ms, FA = 120°, FOV of 256 × 256 × 192 mm^3^ with a voxel size of 0.8 mm^3^.

### 2.4 AMI3 functional MRI acquisition

Six hundred resting-state dynamic scans were acquired using a 2D simultaneous multi-slice echo gradient echo planar sequence with the following parameters: 2.5 × 2.5 mm voxels in-plane; 2.5 mm slice thickness with no gap; 60 transverse slices, FOV = 210 × 210 mm; Matrix = 84 x 84; TR = 700 ms; TE = 30 ms; multiband slice acceleration factor of 6; phase encoding direction Anterior-Posterior; flip angle 53°; bandwidth 2705 hz/pixel; effective echo spacing 0.49 ms. During the functional MRI session, participants were instructed to remain eyes-closed and not to fall asleep.

#### 2.4.1 Longitudinal analysis

The T1w and T2w images at both time points were processed with the Freesurfer 7.2 longitudinal pipeline (Reuter et al., 2012) for automated cortical and subcortical parcellations and tissue segmentation (https://surfer.nmr.mgh.harvard.edu/fswiki/LongitudinalProcessing). The use of T2w images allowed for a more precise segmentation. Briefly, an unbiased within-subject template space and image was created using inverse consistent registration. This longitudinal processing corrects bias created by multiple time points. Then, the following processing steps, such as skull stripping, Talairach transforms, cortical surface reconstruction, cortical atlas registration and subcortical parcellation, were initialized with common information from the within-subject template, significantly increasing reliability and statistical power (Reuter et al., 2012). In particular, we focused on regions of interest (ROIs) from the hippocampal network extracted with the hippocampal subfield segmentation tool of Freesurfer to obtain a head/body/tail subdivision (Iglesias et al., 2015) and from the Brainnetome atlas (Fan et al., 2016) to obtain the following bilateral ROIs at the two time points: Entorhinal cortex, Medial Para-Hippocampal cortex and Lateral Para-Hippocampal cortex. A quality control was performed visually on each ROI. ROIs failing quality were edited manually (non-grey matter voxels were removed) and if they did not pass quality control after editing, they were excluded. The entorhinal cortex did not pass the second quality control after editing for all participants and was therefore excluded from this study.

#### 2.4.2 Resting state analysis

The processing of functional MRI data is described in detail in the supplementary methods. In short, quality control was done using the MRI Quality Control Tool (MRI QC) (Esteban et al., 2017) and preprocessing steps included: fixing gradient distortions and geometric distortions, despiking, intensity normalization, skull-stripping, correcting for physiological noise and movements (with ICA-Aroma) (Pruim et al., 2015). After quality control, one subject lacked some ROIs because of distortions, and another had abnormal movements limiting the analysis to 31 volunteers.

Nilearn (https://github.com/nilearn/nilearn/) was then used to create correlation matrix (with correlation coefficients) between the different ROIs of the Hippocampus and the Para-hippocampal Cortex describe above (contributors et al., 2024).

### 2.5 Statistical analyses

All statistical analyses were performed using R Studio v4.3. Participants’ characteristics were described with mean and standard deviation (SD) for normally distributed variables and median and interquartile range (IQR) for non-normally distributed variables. Associations between demographic variables and variables of interest were tested with sperman correlation for age and education level (package psych, function corr.test) and Student t-test for sex. Cardiovascular effect on variables of interest were tested through proxy variables: with correlation for BMI and Student t-test for hypertension medication.

#### 2.5.1 Longitudinal changes in actigraphic-parameters and MRI volumes

There is a well-known impact of seasons on sleep (Seidler et al., 2023; Yetish et al., 2015) and, to a lesser extent, on rest-activity rhythms (Kume et al., 2017). We took the season of recording into consideration when sleep and rest-activity rhythms were compared between the two time points using two-factors ANOVA with repeated measures (package rstatix, function anova_test). Due to the low number of recordings during Summer and the sample size, we decided to regroup the four seasons in two variables rather than using them separately: “Cold” (Autumn and Winter) and “Warm” (Spring and Summer). Since the objective was to avoid a bias due to seasonality, we did not enter the season of recording in our models but whether our participants had both actigraphy assessments done in the same season (“no season change”) or in different seasons (“season change”). In each ANOVA, the time point variable was used as a “within” participants factor and the season change variable was used as a “between” participants’ factor. Application conditions were respected for each ANOVA: normal distribution of data, independence between observations, homoscedasticity, no outlier. Outlier were removed from the specific analysis in which they were found. In case of an interaction effect between time points and season change, a one-way ANOVA was used to compare the variables between time points by Season change groups. Statistical significance was set at p < 0.05.

Based on the impact of sex on hippocampal network volumes in the first stage of the statistical analyses, we used two-factors ANOVA with repeated measures into consideration when hippocampal network volumes were compared between the two time points. In each ANOVA, the time point variable was used as a “within” participants factor and sex was used as a covariate. Application conditions were respected for each ANOVA: normal distribution of data, independence between observations, homoscedasticity, no outlier. Outlier were removed from the specific analysis in which they were found. In case of an interaction effect between time points and sex, a one-way ANOVA was used to compare the variables between time points by sex groups. Statistical significance was set at p < 0.05.

#### 2.5.2 Prediction of hippocampal formation volumes and functional connectivity

To determine the evolution of a parameter between the two time points (TP1 and TP2), the symmetrized percent change (SPC) was used. It controls the rate of change by the average between the two time points to represent the evolution of a parameter that can be comparable between subjects. Its formula is: SPC = $\frac{rate}{average}\times 100$ where rate = $\frac{TP2-TP1}{time\;interval}$. SPC was calculated for every actigraphic parameter that significantly changes between the two time points.

A linear-quadratic model was used in an exploratory analysis to test if the SPC of sleep and rest-activity rhythms parameters were associated with the hippocampal network volumes and the functional connectivity matrixes at follow-up. For the volumes, only those that significantly changed between the two time points were used as a dependent variable. All variables were scale to z-scores and significance level was set at p < 0.05 after FDR correction for multiple comparisons. Covariables were sex and season change.

Supplementary confounding effects analysis.

Because of the importance of APOE ε4 alleles on AD development (Jack et al., 2015; Nadal et al., 2020) and its impact on hippocampal atrophy (Gorbach et al., 2020), we repeated our analysis in a subsample excluding participants with at least one APOE ε4 allele, leaving a subsample of 29 persons. Significance level was set at p < 0.05 and covariables were sex and season change.

## 3 Results

### 3.1 Participants’ characteristics

Demographic parameters at the two time points are presented in Table 2. The percentage of women in the sample was 40.0% and the distribution in education levels was as follows: 27.3% primary school or less; 24.2% secondary school; 48.5% high school or more. Forty percent of the whole sample took hypertension medication (n = 13).

**Table 2:** Participants’ characteristics.

Demographics (N = 33)	Time Point 1	Time Point 2	Difference
	Mean ± SD	Mean ± SD	FDR
Age, years	75.2 ± 2.4	81.5 ± 2.5	
MMSE^a^, score	28.0 ± 2.0	28.0 ± 1.3	0.637
BMI^b^, kg/m	25.8 ± 4.2	25.7 ± 4.3	0.283
Self-reported sleep Apnea, %	3.0 (1)	9,1 (3)	Ø
Self-reported sleep Medications, %	9.1 (3)	6,1 (2)	Ø
Actimetry recording, days	6.6 ± 0.9	7.0 ± 0.4	**0.038**
“Cold” seasons, %	60.6 (20)	93.9 (31)	**0.008**

Abbreviations: MMSE, Mini-Mental State Examination; BMI, Body Mass Index.

Ø counts too low for calculation.

a Missing data for one subject.

b Missing data for two subjects.

There was an effect of sex on IS and on hippocampal volumes at both time points (p < 0.05). There was no effect of age, education nor BMI on the variables of interest (actigraphic and imaging data) and no difference between those who took hypertension medication and those who did not. Neither MMSE scores nor BMI significantly changed between the two time points while actigraphic assessments were slightly longer and for the most part of them were done during the “Cold” seasons.

### 3.2 Longitudinal changes

#### 3.2.1 Sleep

None of the sleep parameters changed between the two time points and with no impact of season change (Table 3).

**Table 3:** Sleep variables between the two time points with potential season change effect.

Sleep Variables	Time Point 1 Mean ± SD	Time Point 2 Mean ± SD	ANOVA		
			F-value of Time effect	F-value of Season Change effect	F-value of Time x Season Change effect
TIB, hours	8.8 ± 0.8	8.8 ± 1.1	0.759	1.991	0.180
TST, hours	7.1 ± 1.0	7.2 ± 1.0	0.176	0.006	0.521
WASO, hours	1.2 ± 0.7	1.1 ± 0.6	2.062	0.213	0.075
SE, %	80.2 ± 7.2	82.0 ± 5.6	1.599	0.132	0.321
SF, %	32.2 ± 10.9	32.0 ± 9.8	0.000	1.488	3.614

Abbreviations: F-value, Fischer statistic; SE, Sleep Efficiency; SF, Sleep Fragmentation; TIB, Time In Bed; TST, Total Sleep Time; WASO, Wake After Sleep Onset.

### 3.3 Rest-activity rhythms

The ANOVA revealed a decrease in RA (F (1,30) = 5.87; p = 0.022) and an increase in IV (F (1,29) = 4.36; p = 0.046) between the two time points. In addition, we observed an effect of season change on IS (F (1,29) = 4.84; p = 0.036) as well as a time∗season change interaction effect (F (1,29) = 9.48; p = 0.005). To investigate the interaction effect for IS, a one-way ANOVA was performed. It revealed an effect of time on the no season change group (F (1,17) = 5.30, p = 0.034, Δ = - 0.046) meaning a decrease in IS, while the group with a season change did not significantly change between time points (F (1,12) = 4.06, p = 0.067, Δ = + 0.053).

Considering season, rest-activity rhythms parameters significantly degraded over time for each group (season change and no season change) (Table 4).

**Table 4:** Rest-activity rhythms variables between the two time points with potential season change effect.

Rest-activity variables	Season Change	Time Point 1	Time Point 2	ANOVA		
		Median ± IQR	Median ± IQR	F-value of Time effect	F-value of Season Change effect	F-value of Time x Season Change effect
RA	No	0.929 ± 0.030	0.907 ± 0.041	5.87∗	7.09∗	0.31
	Yes	0.897 ± 0.023	0.888 ± 0.043			
IV	No	0.756 ± 0.115	0.828 ± 0.184	4.36∗	0.01	0.02
	Yes	0.776 ± 0.152	0.827 ± 0.146			
IS	No	0.710 ± 0.071	0.666 ± 0.096	0.30	4.84∗	9.48∗∗
	Yes	0.611 ± 0.118	0.665 ± 0.81			

∗p < 0.05; ∗∗p < 0.01.

Abbreviations: F-value, Fischer statistic; IQR, Inter Quartile Range; IS, Inter-daily Stability; IV, Intra-daily Variability; RA, Relative Amplitude.

### 3.4 Hippocampal formation

Due to the significative interaction effect of time and sex on “HPC body L”, a one-way ANOVA was done on each sex groups. It revealed an effect of time on the women group (F (1,12) = 5.73, p = 0.034, Δ = - 20 mm^3^) and an effect of time on the men group (F (1,19) = 33.5, p < 0.001, Δ = - 47 mm^3^). The hippocampal formation and the lateral para-hippocampal cortex volumes significantly decreased between the two time points with the highest effect observed for the right hippocampus (Table 5).

**Table 5:** Hippocampal formation volumes at the two time points.

Volumes (mm^3^)	Time Point 1	Time Point 2	ANOVA		
	Mean ± SD	Mean ± SD	F-value of Time effect	F-value of Sex	F-value of Time x Sex
HPC L	2929 ± 272	2851 ± 298	27.2∗∗∗	2.3	1.2
HPC head L	1456 ± 151	1426 + 157	12.2∗∗	3.1	0.6
HPC body L	984 ± 101	948 ± 115	37.2∗∗∗	2.4	4.4∗
HPC tail L	488 ± 67	476 ± 71	11.7∗∗	0.0	0.4
HPC R	3042 ± 306	2918 ± 325	86.3∗∗∗	5.7∗	0.7
HPC head R	1508 ± 162	1456 + 168	49.0∗∗∗	3.3	0.2
HPC body R	1006 ± 162	956 ± 119	71.8∗∗∗	7.0∗	0.0
HPC tail R	528 ± 66	506 ± 70	30.0∗∗∗	5.0∗	2.8
ParaHPC lat. L	729 ± 146	686 ± 144	23.1∗∗∗	1.4	2.9
ParaHPC med. L	529 ± 76	530 ± 72	0.0	1.3	0.0
ParaHPC lat. R	642 ± 141	615 ± 139	15.4∗∗∗	0.4	4.1
ParaHPC med. R	576 ± 128	565 ± 112	1.7	0.8	0.0

∗p < 0.05; ∗∗p < 0.01; ∗∗∗p < 0.001.

Abbreviations: F-value, Fischer statistic; HPC, Hippocampus; L, Left; lat, lateral; med, medial; R, Right; SD, Standard Deviation.

### 3.5 Rest-activity rhythms evolution predicts hippocampal volumes

Due to the impact of sex and seasonal change on the variables of interest, each model was adjusted for sex and seasonal change. Age had no impact on neither actigraphic nor imaging data; thus, it was not added to the models.

**Fig. 1:**
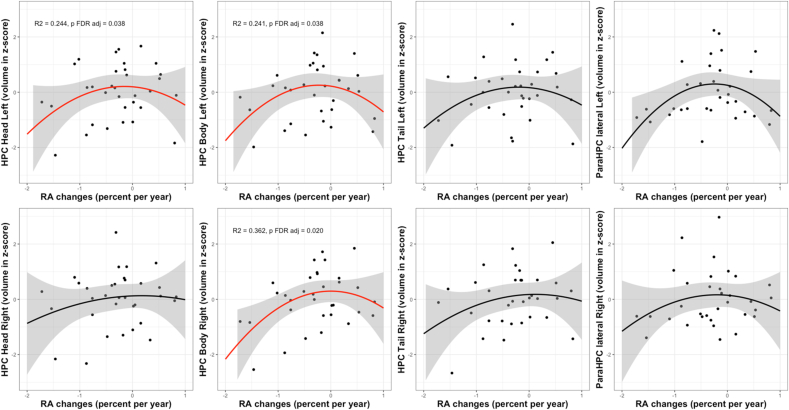
Quadratic associations between the evolution of RA (% of change per year) and the volumes at follow-up of different subparts of the hippocampal network (head, body and tail of the hippocampus and para-hippocampal cortex). For volumes, axis units are z-scores. The quadratic regression line is in red for significant relationship and in black for the others, the 0.95 confidence interval is in grey.

We observed a significant quadratic association between RA changes over time and the volumes of the whole left hippocampus (ß = −0.885, R^2^ = 0.221, F(4,28) = 3.36, p FDR adj = 0.043), left hippocampus head (ß = −0.762, R^2^ = 0.244, F(4,28) = 3.58, p FDR adj = 0.038) and left hippocampus body (ß = −0.897, R^2^ = 0.241, F(4,28) = 3.53, p FDR adj = 0.038) as well as the right hippocampus body (ß = −0.761, R^2^ = 0.362, F(4,28) = 5.55, p FDR adj = 0.020) (Fig. 1).

**Fig. 2:**
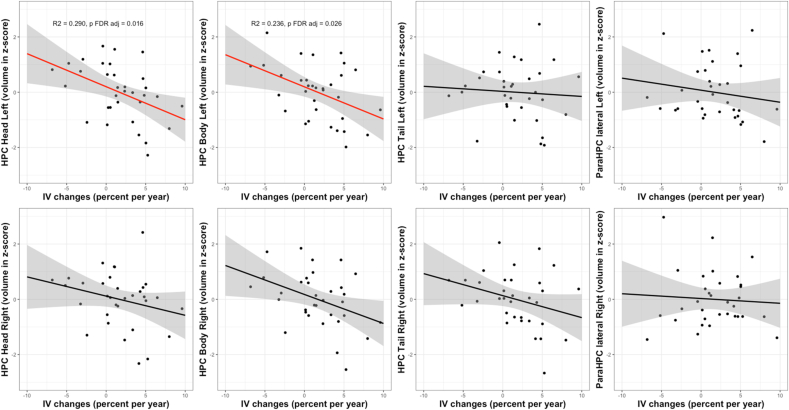
Linear associations between the evolution of IV (% of change per year) and the volumes at follow-up of different subparts of the hippocampal network (head, body and tail of the hippocampus and para-hippocampal cortex). For volumes, axis units are z-scores. The linear regression line is in red for significant relationship and in black for the others, the 0.95 confidence interval is in grey.

Linear models also revealed a negative relationship between IV changes and hippocampus volumes at follow-up (Fig. 2). Increased IV values over time was related to low volumes of hippocampus left (ß = −0.099, R^2^ = 0.207, F(3,29) = 3.78, p FDR adj = 0.035), hippocampus head left (ß = −0.104, R^2^ = 0.290, F(3,29) = 5.35, p FDR adj = 0.016) and hippocampus body left (ß = −0.101, R^2^ = 0.236, F(3,29) = 4.30, p FDR adj = 0.026) at follow-up.

**Fig. 3:**
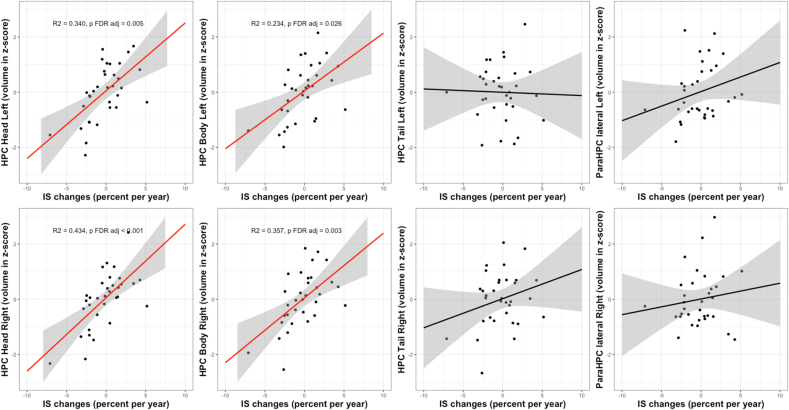
Linear associations between the evolution of IS (% of change per year) and the volumes at follow-up of different subparts of the hippocampal network (head, body and tail of the hippocampus and para-hippocampal cortex). For volumes, axis units are z-scores. The linear regression line is in red for significant relationship and in black for the others, the 0.95 confidence interval is in grey.

Finally, we observed a positive linear association between IS changes and the volume of the left and right hippocampus (β = 0.183, R^2^ = 0.216, F(3,29) = 3.94, p FDR adj = 0.030; β = 0.190, R^2^ = 0.385, F(3,29) = 7.69, p FDR adj = < 0.001, respectively). In particular, a decrease in IS values over time was linked to low volumes of the left and right hippocampus head (β = 0.214, R^2^ = 0.340, F(3,29) = 6.50, p FDR adj = 0.005; β = 0.201, R^2^ = 0.434, F(3,29) = 9.17, p FDR adj <0.001) and body (β = 0.180, R^2^ = 0.234, F(3,29) = 4.27, p FDR adj = 0.026; β = 0.201, R^2^ = 0.357, F(3,29) = 6.92, p FDR adj = 0.003) at follow-up (Fig. 3).

There was no significant relationship between rest-activity rhythms evolution and the volumes of the lateral para-hippocampal cortex.

### 3.6 Rest-activity rhythms evolution and hippocampal formation functional connectivity

There was no significant relationship between rest-activity rhythms evolution and the functional connectivity between the Hippocampal network ROIs in a model adjusted for sex and seasonal change.

### 3.7 Sensitivity analyses

In the subsample of participants without APOE e4 allele, the link between RA and the left hippocampus (ß = −1.045, R^2^ = 0.197, F(3,24) = 2.72, p = 0.054), left hippocampus head (ß = −0.883, R^2^ = 0.191, F(3,24) = 2.65, p = 0.058), left hippocampus body (ß = −1.022, R^2^ = 0.201, F(3,24) = 2.77, p = 0.051) are trends. But RA was significantly linked to the right hippocampus (ß = −0.802, R^2^ = 0.287, F(3,24) = 3.82, p = 0.015), the right hippocampus head (ß = −0.661, R^2^ = 0.260, F(3,24) = 3.46, p = 0.023) and the right hippocampus body (ß = −0.936, R^2^ = 0.343, F(3,24) = 4.65, p = 0.006).

The link between the left hippocampus head and IV evolution (ß = −0.119, R^2^ = 0.120, F(3,25) = 3.33, p = 0.036) as well as IS evolution (β = 0.240, R^2^ = 0.223, F(3,25) = 1.93, p = 0.026) were still significant while other links observed previously for IS and IV were marginally significant.

## 4 Discussion

This exploratory study assessed the longitudinal deterioration of both sleep and rest-activity rhythms parameters with older age, and whether these degradations were linked to hippocampal network impairment. In this population of cognitively normal older people, changes of amplitude as well as a decrease in stability and an increase in fragmentation of the rest-activity rhythms were associated with lower volumes of the hippocampus, in particular the head and the body.

Concerning the evolution of their sleep, we did not find any degradation of objective sleep characteristics between the two time points (7 years apart). This is in accordance with the results of the meta-analysis of Ohayon et al. which reports that the sleep of people over 60 years of age do not change except for SE (Ohayon et al., 2004). In contrast, we observed a degradation of the rest-activity rhythms quality in this population, including a decrease in amplitude (RA) and stability (IS) as well as an increase in fragmentation (IV). Our findings align with those of Li et al. (2020) regarding RA and IV. This agreement extends to IS when accounting for seasonal changes. However, our analyses and the results from Li et al. are not in accordance with actigraphic cross-sectional studies, in which age effect on IS values is either non-existent or positive, i.e. a higher IS with older age (Luik et al., 2013; Musiek et al., 2018). An assumption is that people of different ages or generations could differ in factors other than age (such as way of life, psychological traits, physical robustness, etc. …), that could in turn contribute to different levels of rest-activity rhythms stability. Moreover, in terms of actigraphy-derived sleep characteristics, our population seems to have a preserved sleep compared to other older populations. They have a longer TST, a higher SE and a lower WASO than the Study of Osteoporotic Fractures (SOF) cohort (Blackwell et al., 2006) and the Osteoporotic Fractures in Men Study (MrOS) cohort (Blackwell et al., 2014). Even if we observed a degradation between the 2 time points in terms of rest-activity rhythms parameters, our population has higher stability and lower fragmentation than others cohorts of the same age (Huang et al., 2002; Li et al., 2020), suggesting also a preserve rest-activity rhythms. It is important to note that our population lives in a rural area. Few studies looked at the sleep difference between urban and rural population. It seems that there is a longer TST in rural populations that could be related to more outdoor work, more daylight exposure or possibly a less stressful lifestyle (Ursin et al., 2005). Their living condition could explain the relatively preserved rest-activity rhythms observed.

As expected, we observed an atrophy of the hippocampal formation between the two time points. This is in accordance with several studies showing a negative age effect on the hippocampus volume (Nadal et al., 2020; Nyberg et al., 2022). However, the hippocampus is not a uniform structure and the different hippocampal subfields can evolve differently, with a greater vulnerability of CA1 to age (Flores et al., 2015). In our population, effect sizes were greater for the right sided hippocampus and regarding the subdivisions, size effects were greater for the body followed by the head then the tail of the hippocampus. Likewise, in a longitudinal study of 292 cognitively healthy older people, a greater age effect on hippocampus head and body was observed compared to the hippocampus tail (Gordon et al., 2013).

The goal of this exploratory study was to assess the potential link between age-related rest-activity rhythms evolution and the structural and functional integrity of the hippocampal formation. We found that a worsening of the rest-activity rhythms quality over time was related to low anterior hippocampal volumes at follow-up. Indeed, an increase in fragmentation and a decrease in stability were both linked to low volumes of left anterior hippocampus, and a decrease in stability was also associated with low right anterior hippocampal volume. In addition, RA changes were quadratically related to low volumes of the left anterior hippocampus and the right hippocampus body. The zenith of the quadratic curve is located at the zero z-score for RA evolution, meaning the participants who had no change in RA have the biggest hippocampal volumes and a change, no matter its direction, correlated with a smaller volume. Moreover, there was no significant relationship between hippocampal volumes evolution and rest-activity rhythms parameters at follow-up or between hippocampal volumes evolution and rest-activity rhythms evolution (data not shown), strengthening the possible predictive value of the rest-activity rhythms evolution for adverse brain features. We did not find a relation between rest-activity rhythms evolution and the functional connectivity of the hippocampal network. This could be due to the low spatial resolution of resting state fMRI. Resting state fMRI provides valuable insights into the intrinsic connectivity of the hippocampal network but complementary studies combining both resting state and task-based fMRI could detect specific activations and provide more information on this potential relationship.

Our results add to a growing literature linking the age-related deterioration of rest-activity rhythms to less favorable brain aging features. Some studies showed the predictive ability of rest-activity rhythms parameters at baseline on dementia prevalence at follow-up (Posner et al., 2021; Rogers-Soeder et al., 2018; Tranah et al., 2011; Walsh et al., 2014). In the one study that considered age-related changes of rest-activity rhythm (Li et al., 2020), global cognition changes were positively correlated with longitudinal changes in RA and IS, and negatively correlated with longitudinal changes in IV.

An increase in IV, a decrease in IS as well as changes in amplitude (RA) (regardless of their direction), could be a sign of a dysregulation of biological circadian cycles such as a decrease of the sensitivity or efficiency of the master clock located in the suprachiasmatic nucleus (SCN) [53,54]. In turn, this degradation of the rest-activity rhythms could impact brain health through a perturbation of the 24h rhythm of the glymphatic system, the brain cleaning system able to extract metabolites from the brain (Hablitz et al., 2020) without major perturbation through sleep. This clearance system has been link to the accumulation of β-amyloid and Tau proteins (Iliff et al., 2014; Xie et al., 2013), two lesions hallmarks of AD. Moreover, hippocampal atrophy, one of the most reliable predictor of AD (Bernard et al., 2014; Jack et al., 1998, 2013, 2015) has been related to Tau accumulation in the temporal cortex in TEP imaging studies (Chen et al., 2024; Krishnadas et al., 2022; Park et al., 2019). Age-related grey matter volume loss is also related to cell shrinkage and degeneration of the dendritic network (Blinkouskaya et al., 2021). According to the synaptic homeostasis hypothesis of sleep function, the plasticity of the dendritic network relies in part on the rest-activity rhythms (Raven et al., 2018; Tononi and Cirelli, 2014). A dysregulation of the alternance of periods of wake (with an increasing synaptic load) and sleep (with a synaptic downscaling) could lead to a loss of plasticity ability of the dendritic network. The preservation of the rest-activity rhythms plasticity of the hippocampus could be a crucial component in the maintenance of memory performances in elderly and its loss could lead to AD pathology.

Our study has several limitations. First, due to the longitudinal context and the baseline age of our participants, the total number of individuals is low, limiting therefore the statistical power of our analyses. This prevented the use of additional covariates such as physical activity, lifestyle factors or comorbidities. Second, there was a change of scanner and resolution in between the time points even though it was taken into consideration in the FreeSurfer longitudinal pipeline. Third, sleep was assessed with the proxy of actigraphy and not with a more thorough examination like polysomnography. Lastly, our population is particular in its living situation and cognitive health, which could limit generalization.

However, actigraphy is an easy-to-use and low-cost technique that allows not only the monitoring of sleep-like state but also the rest-activity rhythms. Implementing actigraphic monitoring in clinical settings could help health professionals in detecting individuals at risk for neurodegeneration based on sleep-wake quality. Finally, our study investigates the link between rest-activity rhythms evolution across aging and hippocampal integrity in cognitively normal individuals, as we removed the participants suffering from AD or other neurological disorders, leaving room for further studies that would investigate more specifically the link between rest-activity rhythms, hippocampal integrity and cognitive impairment.

## CRediT authorship contribution statement

**Aurore Jouvencel:** Writing – original draft, Visualization, Formal analysis, Data curation. **Bixente Dilharreguy:** Writing – review & editing, Supervision, Data curation, Conceptualization. **Marion Baillet:** Writing – review & editing, Data curation. **Karine Pérès:** Writing – review & editing, Project administration, Methodology, Funding acquisition, Conceptualization. **Jean-François Dartigues:** Writing – review & editing, Project administration, Funding acquisition, Conceptualization. **Hélène Amieva:** Writing – review & editing, Project administration, Funding acquisition, Conceptualization. **Willy Mayo:** Writing – review & editing, Project administration, Investigation, Funding acquisition, Conceptualization. **Gwenaëlle Catheline:** Writing – review & editing, Supervision, Project administration, Methodology, Investigation, Funding acquisition, Conceptualization.

## Data availability

The data underlying this article cannot be shared publicly to respect the privacy of individuals that participated in the study. The data will be shared on reasonable request to the corresponding author.

## Funding

The 10.13039/100028952AMI study is funded by AGRICA (Association pour la Gestion des Retraites pour le Compte des Institutions Complémentaires Agricoles), MSA (Mutualité Sociale Agricole) de Gironde, 10.13039/501100009429CCMSA (Caisse Centrale de la MSA), 10.13039/501100015721CNSA (Caisse Nationale de Solidarité pour l’Autonomie), 10.13039/501100009243DGOS (Direction Générale de l’Offre de Soins). The AMImage2 project was supported by the 10.13039/501100009243DGOS (Direction Générale de l’Offre de Soin). The AMImage3 project was funded by the association France Alzheimer & Maladies Apparentées.

This study was conducted in the framework of the University of Bordeaux's France 2030 program RRI "IMPACT" that received financial support from the French government.

## Declaration of competing interest

None of the authors have a conflict of interest.

## Data Availability

Data will be made available on request.
