# Supplementary material for: Age-related disturbances in rest-activity rhythms and integrity of the hippocampal network: An exploratory study
Source: Neurobiol Sleep Circadian Rhythms. 2024 Dec 26;18:100111. doi: 10.1016/j.nbscr.2024.100111 (PMC11743803; doi:10.1016/j.nbscr.2024.100111)
Supplement: Multimedia component 1 [file mmc1.docx]

Supplementary Methods

*Quality Control*

A quality control of the functional MRI data was performed using MRIQC software [1]. In particular, subjects exhibiting a mean Framewise Displacement value greater than 0.5mm, a mean DVARS value greater than 0.5% BOLD change or 20% of the scans with FD > 0.5 mm were excluded for excessive motion. In our fMRI analysis, two subjects were excluded limiting the analysis to 31 volunteers.

*Preprocessing*

All functional images were corrected for gradient non linearity effect using gradunwarp (version 7.4.1) and corrected for susceptibility induced distortions using topup method [2,3] with FSL6.0 (FMRIB, Oxford, UK). Then, the images were motion-corrected using linear transformations with MCFLIRT in FSL, intensity-scaled to 1000 and ICA-AROMA was applied to remove motion-induced signal variations that cannot be suppressed with rigid-body registration [4]. ICA-AROMA uses an Independent Component Analysis combined with four spatial and temporal criteria (fraction of the component in the edge of the brain, fraction of the component associated with cerebrospinal fluid within the brain, fraction of high frequencies in the component time-course, correlation of the component time-course with realignment parameters obtained from the rigid-body correction) to identify components representing motion artifacts. Furthermore, a physiological noise correction is applied using the PhysIO toolbox [5] in MATLAB v9.7.0. Briefly, finger pulse oximeter and pneumatic belt were used to respectively record cardiac and respiratory signal during the fMRI acquisition which were processed to model cardiac and respiratory responses that can be regressed out from the fMRI time-series. These physiological regressors consist in low-order Fourier time-series (6 based on cardiac phase, 8 based on respiratory phase, 4 based on an interaction of cardiac and respiratory phase) created using the RETROICOR algorithm [6] and cardiac and respiratory responses based on heart rate variability and respiratory volume per time respectively.

Nuisance regression and band-pass filtering (0.008 Hz - 0.15 Hz) were simultaneously performed using Denoiser toolbox (<https://github.com/arielletambini/denoiser>).

Slice-timing correction was skipped because of the short TR used. Also, no spatial smoothing was applied to the data. Due to the controversy in the interpretation of the functional connectivity results using global signal regression, we did not perform this step in the preprocessing pipeline [7].

*Functional connectivity*

To build the functional connectivity of each subject for the hippocampal formation, Nilearn package was used [8] with the ROIs defined in the volumetric analysis. The binary mask of each ROI was registered to the fMRI data using affine transformations calculated from the registration of the T1-weighted image on a median image of the fMRI dynamic scans using epi_reg (<https://fsl.fmrib.ox.ac.uk/fsl/fslwiki/FLIRT>). Then, the mean fMRI time-series of voxels within each anatomical ROI were extracted. Finally, a Pearson’s correlation coefficient was calculated between these time courses for each pair of the parcellated regions representing the intensities of brain functional connections among the ROIs. A visual quality check was performed which did not reveal any major registration inaccuracy.

1. Esteban O, Birman D, Schaer M, Koyejo OO, Poldrack RA, Gorgolewski KJ. MRIQC: Advancing the automatic prediction of image quality in MRI from unseen sites. *PLoS One*. 2017;12(9):e0184661. doi:10.1371/journal.pone.0184661

2. Andersson JLR, Skare S, Ashburner J. How to correct susceptibility distortions in spin-echo echo-planar images: application to diffusion tensor imaging. *Neuroimage*. 2003;20(2):870-888. doi:10.1016/S1053-8119(03)00336-7

3. Smith SM, Jenkinson M, Woolrich MW, et al. Advances in functional and structural MR image analysis and implementation as FSL. *Neuroimage*. 2004;23 Suppl 1:S208-219. doi:10.1016/j.neuroimage.2004.07.051

4. Pruim RHR, Mennes M, van Rooij D, Llera A, Buitelaar JK, Beckmann CF. ICA-AROMA: A robust ICA-based strategy for removing motion artifacts from fMRI data. *Neuroimage*. 2015;112:267-277. doi:10.1016/j.neuroimage.2015.02.064

5. Kasper L, Bollmann S, Diaconescu AO, et al. The PhysIO Toolbox for Modeling Physiological Noise in fMRI Data. *Journal of Neuroscience Methods*. 2017;276:56-72. doi:10.1016/j.jneumeth.2016.10.019

6. Glover GH, Li TQ, Ress D. Image-based method for retrospective correction of physiological motion effects in fMRI: RETROICOR. *Magn Reson Med*. 2000;44(1):162-167. doi:10.1002/1522-2594(200007)44:1<162::aid-mrm23>3.0.co;2-e

7. Murphy MC, Huston J, Jack CR, et al. Measuring the characteristic topography of brain stiffness with magnetic resonance elastography. *PLoS One*. 2013;8(12):e81668. doi:10.1371/journal.pone.0081668

8. contributors N, Chamma A, Frau-Pascual A, et al. nilearn. Published online January 2024. doi:10.5281/zenodo.10579570
